# Transiliac Osteotomy in Surgical Management of Pelvic Post-Traumatic Malunions: A Retrospective Study

**DOI:** 10.1097/MD.0000000000003144

**Published:** 2016-04-01

**Authors:** Shun Lu, Junwei Wu, Baisheng Fu, Jinlei Dong, Yongliang Yang, Maoyuan Xin, Guodong Wang, Tong-Chuan He, Dongsheng Zhou

**Affiliations:** From the Department of Orthopedics (SL, JW, BF, JD, YY, MX, GW, DZ), Shandong Provincial Hospital Affiliated to Shandong University, Jinan, Shandong, China; and Department of Orthopaedic Surgery and Rehabilitation Medicine, The University of Chicago Medical Center, Chicago, Illinois (T-CH).

## Abstract

While uncommon, post-traumatic pelvic malunions present reconstructive challenges and are associated with significant disability and financial burden. A transiliac osteotomy is a surgical technique useful to correct certain types of pelvic fracture malunions, and is only used when the correction of a limb-length discrepancy is the primary goal. This study aims to present our experience with this technique in the treatment of post-traumatic pelvic malunions.

Eight patients who underwent transiliac osteotomies for post-traumatic pelvic malunions at our department from 2006 to 2011 were included in this study. We reviewed the clinical and radiographic results of these patients.

By the time of their last follow-up, all osteotomy sites and iliac bone graft had healed with no evidence of internal fixation failure. Of the 3 patients who complained of preoperative posterior pain, 2 reported an improvement. All 8 patients noted the resolution of their lower back pain. At the time of their final follow-up, 4 patients could walk normally, 2 had a slight limp without a cane, 1 patient used a cane to help with standing and walking, and the final felt limited during ambulation with a cane. Limb-lengthening relative to preoperative measurements was 2.86 cm (2.2–3.0 cm) at the time of the last follow-up. Two patients were able to return to their previous jobs, 4 patients changed their jobs or engaged in light manual labor while the final 2 were able to perform activities of daily living but were unable to participate in work or labor. Three patients reported being “extremely satisfied” with their outcomes, 3 were “satisfied,” and 2 were “unsatisfied.”

A transiliac osteotomy can be used to manage selected cases of post-traumatic pelvic malunions that are unable to be corrected with a traditional release and osteotomy. However, in these cases the correction of limb-length discrepancies should be the primary reconstructive goal.

## INTRODUCTION

The goals of fracture management are to achieve good anatomic healing and a return to normal function. The surgical treatment of pelvic fractures has improved tremendously over the past few decades.^1,2^ However, malunions still occur secondary to associated nonorthopaedic injuries and the failure to perform a timely internal fixation.^3–7^

Although the ideal way to treat a fracture malunion is to reproduce the original fracture and then re-reduce and fix it, this is not consistently possible with pelvic fracture malunions. Fractures of the posterior pelvic ring, especially when accompanied by the vertical displacement of the lateral sacrum or the sacroiliac joint, often heal with a complex deformity. The loss of bony landmarks and the displacement of neurovascular structures makes a local osteotomy difficult and dangerous.^8^ In some cases, restorations are impossible.

The transiliac osteotomy is a surgical technique that has been found to be useful in certain types of pelvic fracture malunions. While it is commonly not the surgeon's first choice in the treatment of malunions, transiliac osteotomies have been used in select cases to correct limb-length discrepancies. Here, we present our experience with this technique.

## METHODS

Permission for this retrospective study was obtained from the Medical Ethics Committee of our Institution. Eight patients who had post-traumatic pelvic malunions managed with a transiliac osteotomy by our department between 2006 and 2011 were included in this study (Table 1). There were 3 males and 5 females. The mean age was 36.38-year old (26−48 years). The mechanism of injury was a fall for 3 patients and a traffic accident for 5 patients. Based on the AO/Orthopaedic Trauma Association Fracture and Dislocation Classification, 1 injury was a Type C1.1, 4 cases were Type C1.2, 1 case was a Type C1.3, and 2 cases were Type C2. Associated injuries included 5 cases with a traumatic brain injury, 2 cases with a chest injury, 5 cases with an abdominal injury, 7 cases with extremity, acetabular or spinal fractures, and 4 cases with open wounds. Following any indicated emergent management, 5 pelvic fractures were managed conservatively and 3 underwent external fixation. The average time between the injury and the transiliac osteotomy was 9.75 months (6–14 months).

**TABLE 1:**
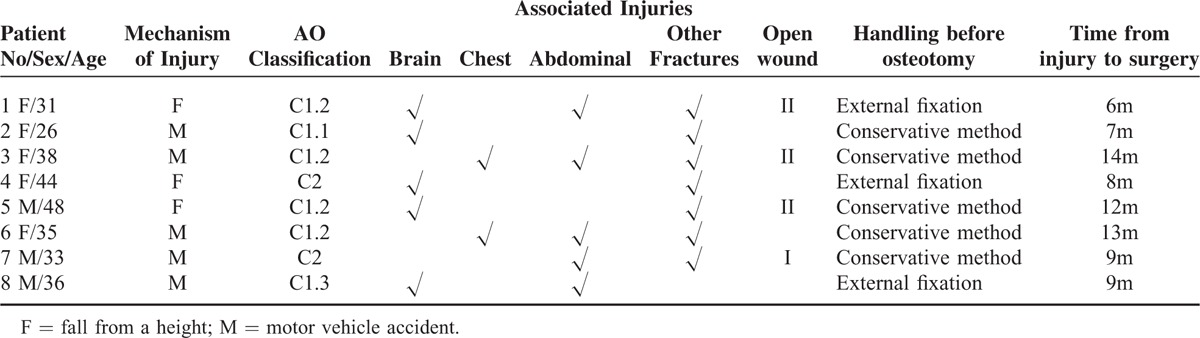
Mechanism of Injury

The inclusion criteria for this study were: a malunion of the posterior pelvic ring in a location where a local osteotomy and release would be technically difficult, unlikely to permit an anatomic reduction or likely to endanger adjacent neurovascular structures; distortion of surgical landmarks; the primary goal of the surgery was to manage a limb-length discrepancy. Those with lumbosacral neurologic deficit requiring further surgery or with fractures amenable to treatment at the fracture site with osteotomy were excluded.

All 8 patients presented with varying degrees of vertical deformity that manifested as a limb-length discrepancy, abnormal gait, and a sitting imbalance. The preoperative limb-length discrepancy, as measured from the umbilicus to the medial malleolus, was 4.06 cm (3.5–5.2 cm) and the average Majeed score 9 was 44.88 points (38−54 points). Of the 8 patients, 3 complained of mild posterior pelvic pain that required daily analgesic use. All patients had lower back complaints such as lumbar fatigue. Lower back pain was most common during weight-bearing activities and was alleviated with rest.

### Operative Technique

In all cases, our initial preferred method was to correct the pelvic malunion with a traditional technique like a 1-, 2-, or 3-stage procedure. A transiliac osteotomy was chosen as the operative technique when restoration with traditional methods was deemed impossible.

When conducting a transiliac osteotomy, a well-measured operative lengthening is crucial. Limb-length discrepancies were measured radiographically as the vertical distance between the inferior border of the L5 (or the superior border of S1) vertebrae to a line connecting the center of the femoral heads. Based upon prior findings, the actual lengthening distance should be less than that of the initial osteotomy gap.^10^ The height of the bone graft should therefore be greater than the intended limb-length correction.

**FIGURE 1:**
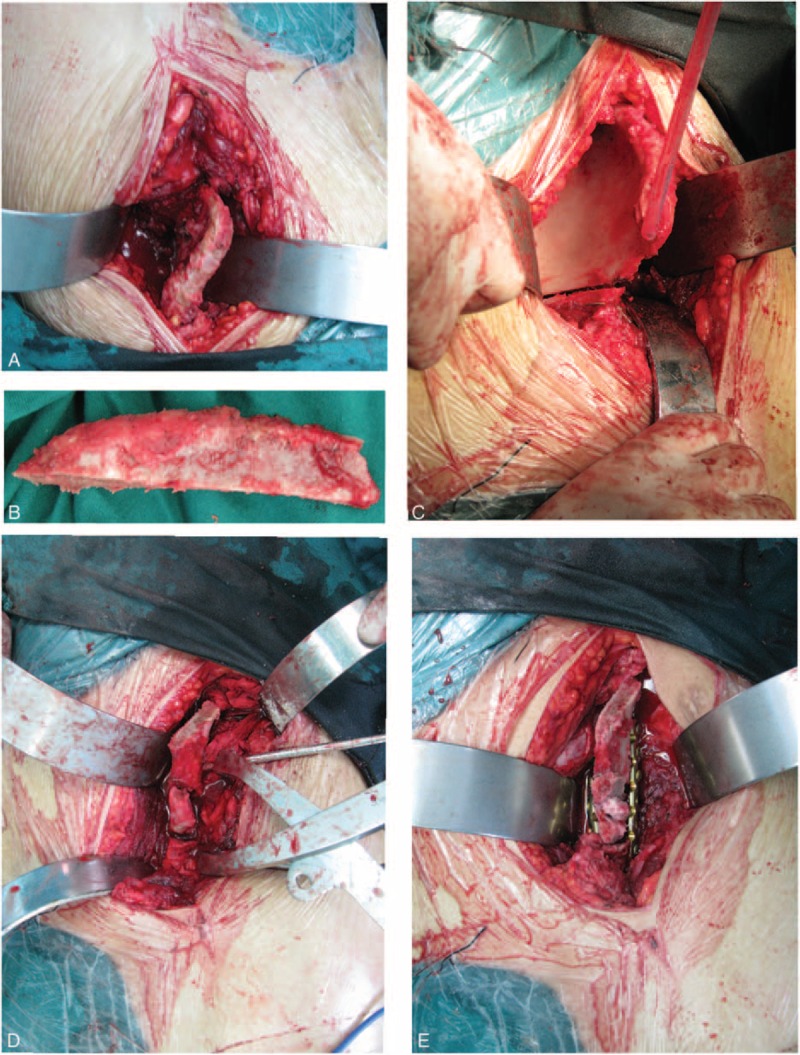
The transiliac osteotomy surgical technique. A, Expose the iliac crest and osteotomy region. B, Take the whole piece of iliac bone as determined during preoperative planning. C, Perform the osteotomy. D, E, Imbed the iliac bone and perform internal fixation.

In our cases, a Smith–Peterson incision was used to expose the iliac crest and the osteotomy, therefore permitting the iliac bone removal and the transiliac osteotomy creation through 1 incision. Taking care to avoid injuring the lateral femoral cutaneous nerve, the iliac bone was excised as determined by our preoperative plan. The osteotomy line ran from the area between the anterior inferior iliac spine and the anterior superior iliac spine to the greater sciatic notch. A pelvic distraction device was then placed, allowing the surgeon to gradually distract the osteotomy site with 1 assistant applying traction to the lower limbs while another fixed the pelvis while protecting the ipsilateral posterior ring. Iliac crest bone graft was placed in contact with both sides of the osteotomy gap. X-ray imaging was used to verify the correct placement of the graft (Figure 1).

The stability of the pelvic ring must be checked intraoperatively. As the transiliac osteotomy does not involve the posterior pelvis, the pelvis remained stable following the osteotomy in all 8 cases. Management of the anterior pelvic ring should only take place after the transiliac osteotomy. However, if the patient is stable after the transiliac osteotomy without any obvious deformity or gapping, anterior ring fixation is not essential as the posterior pelvis provides most of the overall pelvic stability.

### Postoperative Management

All patients began in-bed functional exercises after postoperative pain was well managed, and were permitted to advance to toe-touch weight bearing on the operative side for 6 weeks. Mobilization progressed to 50% partial weight bearing 6 weeks postoperatively, and full weight bearing began 12 weeks after surgery. All mobilization exercises were supervised by a physiotherapist throughout the patient's hospital admission, and were guided by the outpatient and community health services following discharge.

### Follow-Up Assessment

Patients were assessed clinically and radiographically 6 weeks, 12 weeks, 6 months, and 12 months after surgery, with annual follow-ups thereafter. Satisfaction questionnaires and Majeed scores were obtained at each follow-up.

Clinical assessment included patient-reported pain, which included severity, site, duration, and changes with walking or standing. Limb-length discrepancies as measured from the umbilicus to the medial malleolus, sitting imbalances, and overall gait were also evaluated.

Radiographic assessment included routine anteroposterior, inlet, and outlet radiographs of the pelvis to assess changes in limb-length discrepancy, the integrity of the pelvic ring, and healing of the iliac crest bone graft.

Patient satisfaction was divided into 3 levels^4^: highly satisfied, satisfied, and unsatisfied. A highly satisfied patient is one who denies or reports only minimal pain, does not use analgesics, does not need walking aids, and is able to return to normal life and work. A satisfied patient only reports intermittent pain, does not use analgesics, does not need walking aids, and is able to perform all activities of independent daily living. An unsatisfied patient is one with intractable pain that requires daily analgesic use, requires walking aids, and whose daily life and work are restricted. The Majeed score^9^ was also calculated at each follow-up.

## RESULTS

**FIGURE 2:**
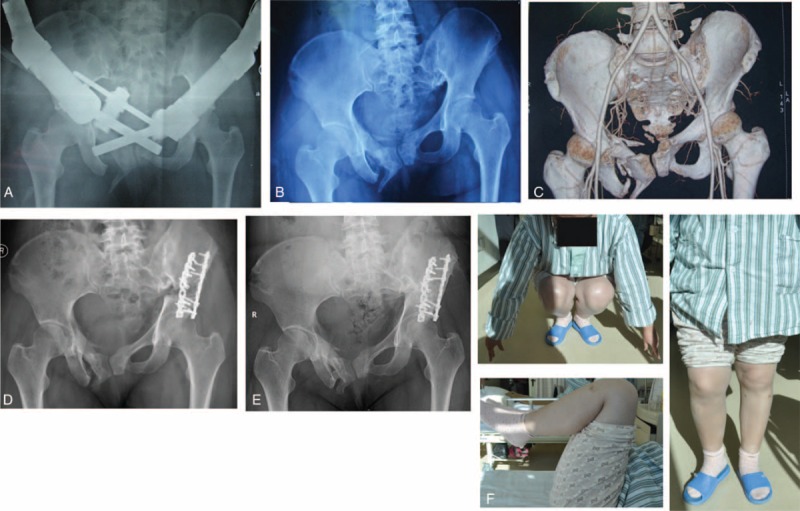
A representative pelvis fracture malunion. A, External fixation after damage control interventions were performed. B, Preoperative anteroposterior X-ray. C, Preoperative 3-dimensional computed tomography reconstruction. D, Anteroposterior X-ray after osteotomy. E, The lower limb function (2 years after surgery). F, The patient's function after the operation.

The mean surgical time was 247.5 min (180–360 min) with a mean blood loss of 2225 mL (1200–4200 mL). The average length of hospitalization was 23.13 days (16–38 days). After a mean follow-up of 23.25 months (12–36 months), all iliac grafts were healed, no osteotomy gap was noted and no internal fixation failure was observed (Figure 2). Of the 3 patients with preoperative posterior pelvic pain, 2 improved. Lower back pain was relieved in all 8 patients. At the final follow-up, 4 patients had a normal gait and 2 had a slight limp without the need for a cane or crutch. Of the remaining 2 patients, 1 used a cane to help with long-term standing and walking, while the other was capable of only limited ambulation with a cane. The average limb lengthening was 2.86 cm (2.2−3.0 cm) at the final follow-up. Two patients returned to their previous work. Four patients changed their jobs or engaged in light manual labor. The other 2 were capable of activities of daily living but were unable to participate in work or labor. Three patients were extremely satisfied, 3 satisfied, and 2 unsatisfied with the outcome of their surgical treatment. The average Majeed score was 60.25 points (44−78 points) (Table 2).

**TABLE 2:**
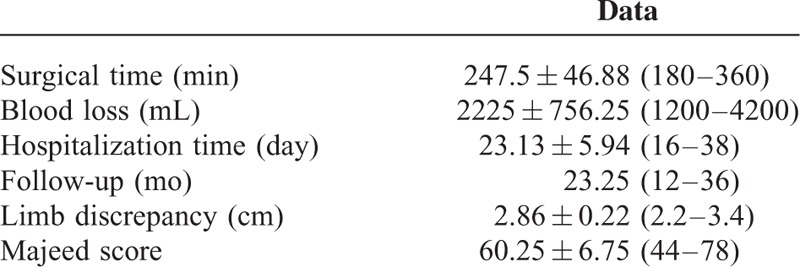
Surgery Characters

Postoperative complications in our patient group included delayed soft tissue healing in 2 patients, which required 3 to 4 weeks to close. Two patients complained of pain around their incision and anterolateral femoral skin numbness and paresthesias. These later resolved in 1 patient 5 months after surgery. One patient was diagnosed with a deep vein thrombosis of the left leg, and was maintained in stable condition with anticoagulation. Cystic lesions were found on the hips and thighs on the operative side of 1 patient, which did not recur following local excision. These lesions were confirmed on pathology as soft tissue cysts with histologic evidence of chronic inflammation and surrounding fibrosis. No sciatic nerve stretch injuries were observed, and no vascular or nerve damage was reported postoperatively.

## DISCUSSION

### Post-Traumatic Pelvic Malunions Are Often Discovered After the Ideal Operative Window Has Elapsed

Unstable pelvic fractures are often accompanied by hemodynamic instability, traumatic brain injuries, and other life-threatening conditions that take operative precedence over orthopaedic injuries. Per the damage control theory, it is inappropriate to perform definitive internal fixation of pelvic fractures when the patient presents with life-threatening injuries. Although pelvic binders and external fixation are the most common choices for managing pelvic fractures in the setting of an unstable patient, these modalities are only temporary solutions and cannot be used as definitive fixation.^2,11^

Ultimately, many patients with pelvic fractures heal with complex deformities due to the inability of the surgeon to perform definitive fracture fixation during the ideal treatment window. Previous studies have reported that the rates of nonunion and malunion are 55% to 75% in vertically unstable pelvic fractures when conservative or external fixation methods are used. The most frequent cause of pelvic malunion or nonunion is inadequate or no treatment.^3,4,12,13^ Of the 8 patients in our study, 5 were treated with conservative methods and 3 were treated with external fixation. All were treated in this manner due to life-threatening injuries, and all local soft-tissue injuries were managed properly. These patients presented an average of 9.75 months after their injuries. We propose that an increased emphasis within damage control orthopaedics be placed on the timely fixation of unstable pelvic fractures, which can significantly reduce the rate of debilitating nonunions and malunions

### Conventional Methods to Treat Malunions Do Not Have a High Success Rate

There are many reports that discuss the management of pelvic malunions and nonunions.^3–7^ Pain, deformity, and gait abnormalities are the major reasons why patients seek a surgical evaluation. Most often, the surgical correction of pelvic malunions and nonunions involves a 1-, 2-, or 3-stage procedure.^3,4,12^ Although some slight deformities can be treated successfully with a 1- or 2-stage operation, a 3-stage procedure is essential for patients with significant deformities. Although surgery can improve the majority of the preoperative symptoms of patients with pelvic deformities, an anatomic reduction may remain impossible in a small subset of patients. Mears and Velyvis^4^ reported on 137 cases of pelvic malunions treated with surgery. The anatomic reduction, general satisfaction, and overall dissatisfaction rates were 50%, 35%, and 15%, respectively. Oransky and Tortora^3^ reported that an anatomic reduction could not be achieved in 21% of patients. Further, the complex and time-consuming reconstruction may cause increased blood loss and a higher complication rate compared with fresh pelvic fractures.^3,4,7^

### Transiliac Osteotomy Is an Alternative to Conventional Methods

**FIGURE 3:**
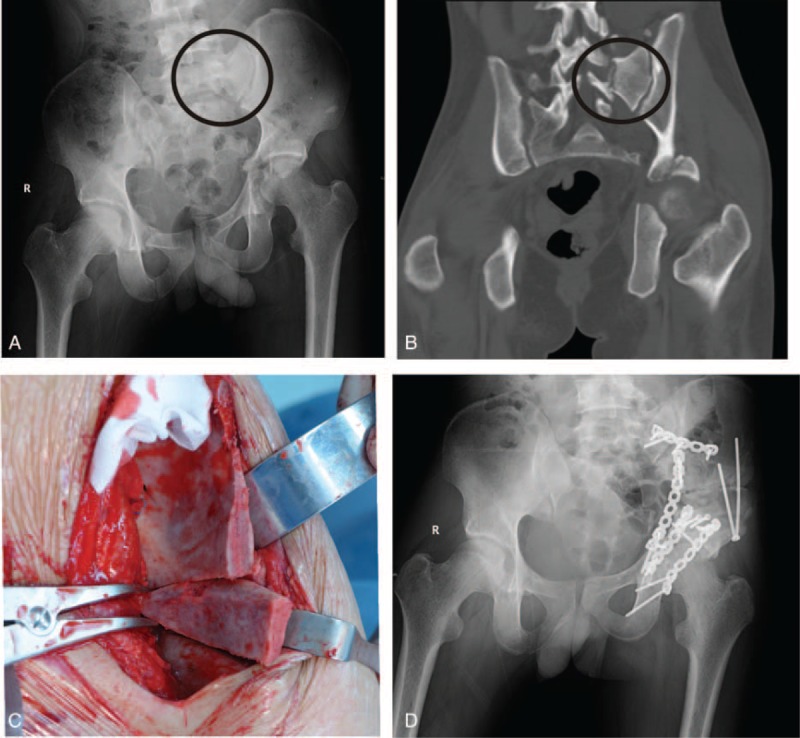
A, B, A pelvic malunion following a Type C1.3 fracture with an accompanying acetabular injury. C, The transiliac osteotomy. D, Reconstruction after surgery.

The transiliac osteotomy is not the first choice for pelvic malunions at our institution. Whenever possible, the best way to treat a malunion is to recreate the fracture at its original location, and then re-reduce and fix it in its anatomic position. But in some older pelvic fractures, this may not be possible or safe. Fractures of the posterior pelvic ring, especially those accompanied by the vertical displacement of the lateral sacrum or the sacroiliac joint, often heal with complex deformities. The loss of bony landmarks and the displacement of neurovascular structures from their normal positions make a local osteotomy difficult and dangerous. The increased risk of significant blood loss, scar contractures, and decreased bone mineral density also complicates traditional reconstruction and fixation. A wide range of osteotomy and release techniques may lead to massive bleeding and a high risk of neurovascular injury. Considering the above factors, we turned to the transiliac osteotomy. In all of our cases, we first attempted the traditional single to multistage technique, resorting to transiliac osteotomies when re-reductions were impossible (Figure 3).

**FIGURE 4:**
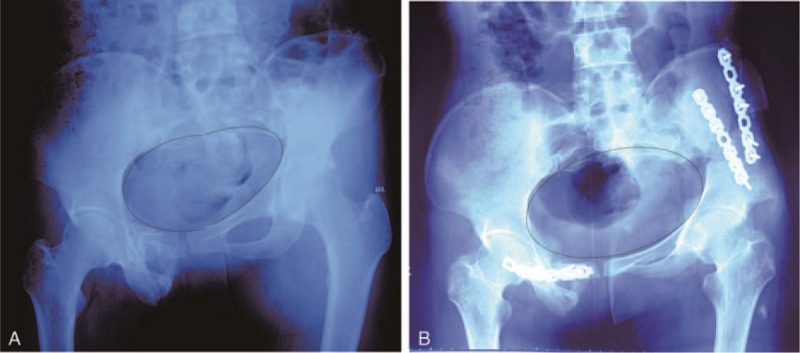
Anterior-posterior X-ray view of the true pelvic geometry. A, The pelvic ring prior to surgery. B, The pelvic ring after surgery.

The transiliac osteotomy is a surgical technique that is useful in certain types of pelvic fracture malunions, and is most often utilized to correct limb-length discrepancies and deformity-related complications. Unstable pelvic fractures often have multiplanar and rotational components. They often combine distraction/impaction and flexion/extension in the x-axis, cephalad/caudad translation, and internal/external rotation in the y-axis, and anterior/posterior translation and abduction/adduction in the z-axis.^14^ In our study, 5 of the 8 patients had internal rotation malunions, 3 patients had external rotation malunions, and all patients had a cephalad translation leading to a limb-length discrepancy and a sitting imbalance. Transiliac osteotomies corrected the limb-length discrepancy in the y-axis, and the downward movement of the inferior ilium shortened the distance between the 2 ischial tuberosities or put them on the same horizon. The limb-length discrepancy and sitting imbalance problems were corrected by surgery. On anterior-posterior X-ray views of the pelvis, the true pelvic geometry was restored from a “pear-shaped” to a semielliptical spherical structure.^15^ However, the oblique diameter of the pelvis enlarged after surgery (Figure 4). Six patients had improved pain symptoms and were back to or close to a normal gait and 2 patients kept a slight limp. Lower back complaints resolved in all patients. Three patients were extremely satisfied, 3 satisfied, and 2 unsatisfied with their surgical treatment outcomes. We have achieved good results in the correction of pelvic fracture vertical malunions with a transiliac osteotomy. However, as the surgery does not involve the posterior pelvis and cannot correct a posterior rotational deformity, we do not recommend that this technique be used on patients with intractable posterior pelvic pain or posterior ring instability.

### Transiliac Osteotomy Operative Technique

The transiliac osteotomy has been used in the treatment of polio sequelae with limb-length discrepancies,^10^ and has been extensively adopted and improved after Mills report.^16^ However, the use of a transiliac osteotomy in the treatment of a pelvic malunion has been rarely reported. When applying this method we find that several factors should be taken into account. A careful preoperative assessment should be performed, and the whole block of the ilium should be taken according to the preoperative assessment. The 3-dimensional structure of the pelvis and the trapezoidal distraction gap should be considered. Further, the height of the bone graft should be greater than the preoperative lengthening goals. The iliac crest bone graft should be obtained anterior to the transiliac osteotomy. These procedures should be performed while minimizing bleeding and ensuring that the patient is in stable condition throughout the case. Intraoperatively, the lateral femoral cutaneous nerve should be protected, and care should be taken to avoid injuring the vessels and nerves near the osteotomy site. The iliac bone should be preprocessed, and during placement the cancellous bone should be in contact with both sides of the osteotomy gap. The ipsilateral posterior ring should be protected and monitored by an assistant.

### Consider Neurovascular Injury When Extending the Incision

The incidence rate of neurovascular injuries associated with pelvic fractures is approximately 10%,^17^ and is higher in unstable pelvic fractures.^6^ It has also been reported that 15.4% of unstable sacral fractures resulted in nerve lesions after operative fixation.^18^ It is widely accepted that the risk of injury to the sciatic nerve during a total hip arthroplasty (THA) is minimal when limb-lengthening is limited to 4 cm.^19^ However, the higher quantity and increased complexity of the nerves of the pelvic area make the fixation of pelvic fractures more challenging than THAs. We therefore recommend limiting limb-lengthening to 3 cm. We had no sciatic nerve stretch injuries or postoperative nerve damage in our case series.

## CONCLUSION

A transiliac osteotomy corrects the limb-length discrepancies of pelvic malunions and places the ischial tuberosities in or near the same horizon. Leg-length discrepancies and sitting imbalance problems are corrected by surgery, and this method can correct the pelvic vertical deformity that is difficult to do with a general release and osteotomy. However, as the surgery does not involve the posterior pelvis and cannot correct a posterior rotational deformity, patients with intractable posterior pain or posterior ring instability should not undergo this procedure. Postoperatively, patients need time to adapt to the changed spatial relationship of their pelvic ring and acetabulum and the impact of the procedure on the soft tissues of the hip, femoral head, and spine. We have successfully corrected pelvic fracture malunions using the transiliac osteotomy. However, because of the typically unstable condition of these patients and the small sample size available in our study, additional high quality trials with larger samples and longer follow-up periods are needed to permit conclusive recommendations.
